# A Comparative Analysis of Botulinum Toxin Use Versus Other Therapies for Temporomandibular Disorders: A Systematic Review

**DOI:** 10.7759/cureus.70389

**Published:** 2024-09-28

**Authors:** Rahaf H Kharraz, Noor A Mushan, Ghadi M Alshehri, Meshari M Dhaen, Heyam A AlGalal, Rim A Khashfa, Mohammed Shammas, Mariam A Bagabas

**Affiliations:** 1 General Dentistry, Almeswak Dental Clinic, Jeddah, SAU; 2 General Dentistry, Ibn Sina National College for Medical Studies, Jeddah, SAU; 3 General Dentistry, Alabeer Medical Company, Jeddah, SAU; 4 General Dentistry, Blue Hills Clinic, Najran, SAU; 5 General Dentistry, Alpha Clinic, Jeddah, SAU; 6 Prosthodontics, Ibn Sina National College for Medical Studies, Jeddah, SAU

**Keywords:** botulinum toxin, electromyographic, maximum mouth opening, occlusal force, pain, temporomandibular disorder

## Abstract

Existing literature regarding the efficacy of Botulinum toxin A (BoNT-A) therapy in improving the clinical outcomes of temporomandibular disorders (TMDs) is ambiguous and lacks consistency. Thus, this study aimed to evaluate the efficacy of BoNT-A in reducing pain, occlusal force, electromyographic (EMG) changes, and maximum mouth opening compared with placebo and other interventions. An electronic database search was conducted using MEDLINE, PubMed, Google Scholar, the Cochrane Library, and ClinicalTrials.gov from January 2000 to June 2024 to identify randomized controlled trials (RCTs). A manual search complemented the electronic search. The Risk of Bias 2 (RoB 2) assessment was used to evaluate the internal validity of the included studies. A total of 1719 studies were identified, of which 23 fulfilled the inclusion criteria. Nineteen of these studies evaluated pain levels (primary outcome) after BoTN-A therapy, with six of them observing a decrease. In terms of secondary outcomes, seven of 10 studies noted an increase in maximum mouth opening, while all six reported a drop in EMG activity, and all four found a decrease in occlusal force following BoNT-A therapy. Muscle activity and biting force were significantly reduced in the therapeutic groups. Clinicians must consider these adverse events before treating patients with BoNT-A therapy. Additional regulatory guidelines and standardization of injection protocols are essential in improving therapeutic outcomes and patient safety. These findings suggest that BoNT-A may be a feasible option for TMD management but should be used with caution in clinical settings.

## Introduction and background

Temporomandibular disorders (TMDs) affect the temporomandibular joint (TMJ) and masticatory muscles, causing symptoms such as orofacial pain, limited mouth opening, joint clicking, and cephalalgia [1]. These symptoms do have a major effect on daily activities and quality of life [2]. TMD is a multifactorial disease, and diagnostic evaluation varies. This can make the diagnosis overly complex, often involving different symptoms, such as facial pain, bruxism, and masticatory muscle hyperactivity. Diagnostic tools include radiographs, orthopantomograms (OPG), and magnetic resonance imaging (MRI) [3].

Interventions for treating TMD include a spectrum of management strategies that can be nonmedicinal or surgical, with a primary focus on conservative methods. It is commonly treated with pharmacologic medications, physical modalities, occlusal splints, psychological treatments, and manual therapy [4-8]. However, these methods may not always provide complete relief.

In recent years, the potential use of Botulinum toxin A (BoNT-A), a powerful neurotoxin produced by *Clostridium botulinum* that has muscle-relaxant and anti-inflammatory actions, as TMD therapy appears to be a significant option for pain relief [9]. BoNT-A injections can reduce hyperactivity in facial muscles, such as the masseter, temporal, and pterygoid muscles, thereby decreasing bruxism events and pain [10].

This systematic review is intended to evaluate the latest evidence from randomized controlled trials (RCTs) on how well BoNT-A relieves TMD symptoms. Particularly, its efficacy in reducing pain as measured by visual analog scale (VAS), occlusal force, electromyographic (EMG) changes, and maximum mouth opening compared to placebo or other interventions. This review aimed to fill the literature gap and provide clinicians with evidence-based treatment options that improve patient outcomes. To accomplish this, an electronic search of databases like MEDLINE, PubMed, Google Scholar, the Cochrane Library, and ClinicalTrials.gov was conducted to detect appropriate randomized controlled trials (RCTs) published between January 2000 and June 2024. Studies were then evaluated for inclusion based on predefined criteria, and the internal validity was evaluated using the Risk of Bias 2 (RoB 2) tool.

## Review

Materials and methods

Study Design

This research was performed following the guidelines of the Preferred Reporting Items for Systematic Reviews and Meta-Analyses (PRISMA).

Study Inclusion and Exclusion Criteria

Studies that fulfilled the inclusion criteria were included in this systematic review. The inclusion criteria were mentioned in the PICOS format (Population, Intervention, Comparison, Outcomes, and Study Design). Population: Adult patients (>18 years) presenting with TMDs; Intervention: BoNT-A injection for pain management; Comparison: Placebo, no treatment, or any other interventions like occlusal splints, drug therapy, or physical therapy; Outcome: Changes in pain level after BoNT-A treatment; Study design: randomized control trials (RCTs) conducted in humans. The literature search was conducted between January 2000 and June 2024. Studies that were excluded include those using BoNT-A for conditions other than TMDs, literature reviews, case reports, commentaries, letters to the editor, and animal and in vitro studies.

Literature Search Strategy

An electronic database search was conducted on MEDLINE, PubMed, Google Scholar, Cochrane Library, and ClinicalTrials.gov from January 2000 to June 2024. The search results involving the use of Medical Subject Headings (MeSH) terms and additional specific search strategies are described in Table 1. Furthermore, the references cited in the identified articles were manually examined to identify additional RCTs.

**Table 1:** Electronic search strategies for MEDLINE, PubMed, Google Scholar, Cochrane Library, and ClinicalTrials.gov

Database	Keywords
MEDLINE/PubMed	((("TMJ") OR ("temporomandibular disorder")) OR ("temporomandibular joint")) OR ("Temporomandibular Joint Disorders")) AND ("botulinum toxin")) OR (BTX OR BTX-A)) OR (Botox)) OR ("Botulinum Toxins")) AND ("myofascial pain")) OR (pain)) AND (Masticatory Muscles)
Google Scholar	“botulinum” AND “temporomandibular" AND “myofascial pain” AND "masticatory muscles”
Cochrane Library	("TMJ syndrome") OR ("temporomandibular joint disorder") OR ("temporomandibular") AND ("Botox") AND ("myofacial pain")"
ClinicalTrials.gov	“botulinum” AND “temporomandibular" AND myofascial pain

Study Selection and Extraction

The EndNote software (Thomson Reuters EndNote X7®; New York, USA) was used as the reference management system, in which all papers identified in the literature search were collected. The process of selecting studies was performed independently in two stages involving two reviewers (RHK and NAM). First Stage: screening titles and abstracts based on an electronic database search. Studies that did not meet the selection criteria and duplicate studies were excluded. Second stage: identification of relevant studies that fulfilled the inclusion criteria. The references were also screened to obtain additional information.

In situations in which there was a disagreement about the study selection, the assistance of a third reviewer (GMA) was sought to reach a mutual consensus. The two reviewers (RHK and NAM) followed a systematic approach to gather information independently. The extracted information was collected using Microsoft Excel 2016 software (Microsoft). The extracted data obtained from the studies included the following details: study design, participant's characteristics, diagnostic criteria for TMD, details of the intervention (dose, muscle, injection site), control/ comparative cohort, assessed outcome, primary and secondary outcome measures, follow-up period, and results summary.

Literature Quality Evaluation

The evaluation of the quality of all studies included in the analysis was performed using the Cochrane Risk of Bias tool (RoB 2.0; Cochrane, London, UK) to evaluate the internal validity of the included systematic reviews. The following domains were considered: randomization process, deviations from the intended interventions, missing outcome data, outcome measurement, and selection of the reported result. Each domain was categorized into three categories: low risk, high risk, and some concerns. Subsequently, an overall risk of bias was assigned to each study based on whether all domains had a low risk, whether there was some concern in at least one domain, or whether one or more domains raised concerns (high risk). Two researchers (RAK and MMA) independently reviewed the selected articles and scored them according to the abovementioned criteria. Any discrepancy was resolved by a third researcher (HAA), who was consulted for input.

Results

**Figure 1:**
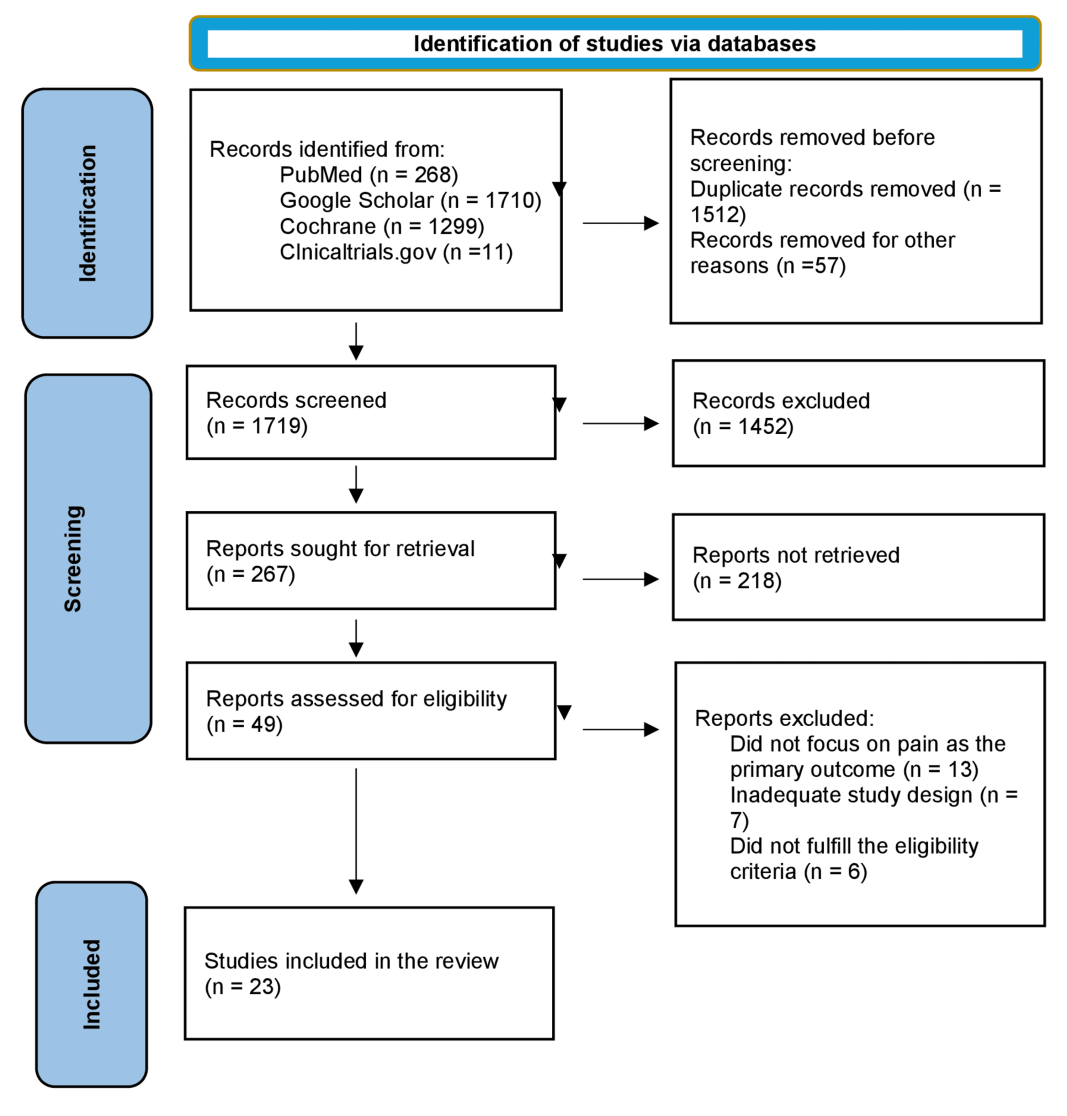
Flowchart of literature search

Figure 1 shows the selection and identification of studies. After the removal of duplicate studies, 1719 studies that underwent title and abstract screening were identified. A total of 49 full-text articles were assessed for eligibility. The 49 articles underwent full-text review, and only 23 studies fulfilled the inclusion criteria. All selected studies were randomized controlled trials (RCTs) with four crossover study designs and 19 parallel study designs.

Study Characteristics

The study characteristics are presented in Table 2.

**Table 2:** Summary of descriptive characteristics of included studies (n=23)

Sl. No.	Author (year)	Study design	Participant characteristics (sample size, age, sex)	Diagnostic criteria for TMD	Intervention (dose, muscle, injection site, Botox)	Control/ comparative cohort	Assessed outcome	Follow-up period
1	Parreira da Silva et al. (2020) [11]	randomized, double-blind study	N= 30; M/F distribution: 0/30	Clinical examination	BoNT-A injections (n=15)	Placebo (n = 15)	EMG	1, 2, 3, 4, 5, and 6 months
2	Sitnikova et al. (2022) [12]	randomized, crossover, controlled study	N = 57; average age: 38.2, M/F distribution: 10/47	Diagnostic Criteria for TMD	BoNT-A injections (n = 57)	Placebo (n = 57)	EMG	2, 11, 16, 18, 27 and 32 weeks
3	Shim et al. (2020) [13]	randomized, double-blind, placebo-controlled, parallel-design study	N = 23; average age: BoNT-A= 32.5, placebo= 28.9, M/F distribution: 10/13	Clinical examination	BoNT-A injections (n = 13)	Placebo (n = 10)	EMG	1 and 3 months
4	Zhang et al. (2016) [14]	randomized	N = 20; average age: BoNT-A= 26, placebo= 28, M/F distribution: 16/4	Clinical examination	BoNT-A injections (n = 10)	Placebo (n = 10)	Occlusal force	1, 3, and 6 months
5	Gupta et al. (2016) [15]	randomized, double-blinded, placebo-controlled Study	N= 24; age range: 20–50 years	Clinical examination	BoNT-A injections (n = 12)	Placebo (n = 12)	Pain	2 and 4 weeks
6	De la Torre Canales et al. (2020) [16]	randomized, controlled study	N = 80; average age: 36.8, M/F distribution: 0/8	Research Diagnostic Criteria for TMD	BoNT-A injections (n = 60)	Placebo (n = 20)	Pain	1, 2, 3, 4 weeks, and 6 months
7	Kurtoglu et al. (2008) [17]	randomized, double-blinded, placebo-controlled, prospective study	N=24; BoNT-A group: average age= 29.6 ± 12.7, age range= 16-53 years, M/F distribution = 2/10; Placebo group: average age= 23.4 ± 4.7, age range= 20-34 years, M/F distribution = 2/10	Research Diagnostic Criteria for TMD	BoNT-A (n = 12)	Placebo (n = 12)	Pain	2 weeks and 1 month
8	Ernberg et al. (2001) [18]	randomized, double-blind, crossover study	N = 21; average age= 38.0, M/F distribution = 2/19	Research Diagnostic Criteria for TMD	BoNT-A injections (n = 21)	Placebo (n = 21)	Pain	1 and 3 months
9	De Carli et al. (2016) [19]	randomized clinical trial	N= 15; average age: 38, M/F distribution = 2/13	Clinical examination	BoNT-A (n=7) 1st injection	Laser (n=8)	Pain	1 month
10	Nixdorf et al. (2002) [20]	randomized, double-blind, placebo-controlled, crossover study	N = 15; average age: 33 years	Research Diagnostic Criteria for TMD	BoNT-A injections (n = 15)	Placebo (n = 15)	Pain	2 months
11	Guarda-Nardini et al. (2008) [21]	double-blind, controlled placebo, randomized study	N = 20; average age: 38 ± 12, M/F distribution = 10/10	Diagnostic Criteria for TMD	BoNT-A injections (N = 10)	Placebo (N = 10)	Pain	1 week, 1 and 6 months
12	Guarda-Nardini et al. (2012) [22]	randomized, controlled study	N = 30; age range: 26-29 years, M/F distribution = 8/22	Diagnostic Criteria for TMD	BoNT-A injections (N = 15)	Fascial manipulation (N = 15)	Pain	3 months
13	Kim et al. (2023) [23]	randomized, double-blind, placebo-controlled, pilot study	N = 21; average age: 33.95 ± 9.70, age range: 21–53 years, M/F distribution = 2/19	Diagnostic Criteria for TMD	BoNT-A injections (N = 14)	Placebo (n = 7)	Pain	1, 2, and 3 months
14	Kütük et al. (2019) [24]	randomized, prospective study	N= 40; average age: 33.8, age range: 20–60 years, M/F distribution: 11/29	Clinical examination	BoNT-A injections (n = 20);	Dry needling technique (n = 20)	Pain	1.5 month
15	Montes-Carmona et al. (2020) [25]	randomized, controlled study	N = 60; average age: BoNT-A= 42.4, placebo= 43, M/F distribution: 28/32	Research Diagnostic Criteria for TMD	BoNT-A injections (n = 20)	Placebo (n = 20)	Pain	1, 2, 3 weeks, and 1, 3, 6 months
16	Ângelo et al. (2023) [26]	double-blind, randomized, controlled study	N= 15; average age: 26.5, age range: 18-39 years, M/F distribution: 1/14	Diagnostic Criteria for TMD	BoNT-A injections (n = 7)	Placebo (n = 8)	Pain	5 weeks and 6 months
17	Rezazadeh et al. (2022) [27]	randomized, double-blind, controlled, study	N = 36; average age: BoNT-A= 28.8, placebo= 24.8, M/F distribution: 17/19	Research Diagnostic Criteria for TMD	BoNT-A injections (n = 18)	Placebo (n = 18)	Pain	1 week, 1 and 3 months
18	Jadhao et al. (2017) [28]	randomized, controlled study	N = 16; TMJ pain and bruxism for > 6 months	Clinical examination	BoNT-A injections (n = 8)	Placebo (n = 8)	Pain	1 week, and 3 and 6 months
19	Patel et al. (2017) [29]	randomized, double-blind study	N = 19; Age not mentioned in the study	Ordinal scale	BoNT-A injections (n = 10)	Placebo (n = 9)	Pain	1, 2, and 3 months
20	Ondo et al. (2018) [30]	randomized, placebo-controlled, parallel-design study	N = 22; average age: 47.4, M/F distribution = 4/18	International Classification of Sleep Disorders	BoNT-A injections (n = 13)	Placebo (N = 9)	Pain	1, 2 months
21	Von Lindern et al. (2003) [31]	randomized, blinded, placebo-controlled study	N = 90; Age not mentioned in the study	clinical examination	BoNT-A injections (n = 60)	Placebo (N = 30)	Pain	1 month
22	Cruse et al. (2022) [32]	double-blind, randomised, placebo-controlled, cross-over study	N = 22; average age: 42.1, M/F distribution: 8/14	International Classification of Sleep Disorders-Revised	BoNT-A injections	Placebo (number not mentioned)	Pain	1, 3 months
23	Al-Wayli H (2017) [33]	prospective, randomized, controlled, parallel-group study	N = 50; average age: 45.5 ± 10.8, M/F distribution = 0/50	International Classification of Sleep Disorders	BoNT-A injections (n = 25)	Placebo N = 25	Pain	3 weeks, and 2, 6, and12 months

Overall, the study population included 750 patients presenting with TMD aged 18 to 60 years. The effect of BoNT-A on pain was assessed in all studies except for four studies [11-14]. Six studies measured changes in muscle activity using EMG [11-13,15-17], 10 studies assessed the maximum jaw opening in participants [18-27], and four studies measured changes in the occlusal force [11,12,14,28]. The follow-up period varied across all studies, with a minimum follow-up period of one week and a maximum follow-up period of 12 months. BoNT-A injections were administered into the facial muscle predominantly in the masseter muscle except in two studies [24,25]. The second most frequently targeted injection site was the temporal muscle [11,12,15-22,25,26,28-32]. Various studies administered BoNT-A into the pterygoid muscle [24,25,27,29,31,32]. The doses of BoNT-A were highly distinct across each study and ranged between 25 units and 150 units on each side (left and right) [13,23]. The primary and secondary findings of the included studies are given below.

Primary Outcome

The outcome assessment is presented in Table 3. 

**Table 3:** Primary and secondary outcomes of included studies (n=23)

Sr no.	Study ID	Primary outcome measure	Primary outcome results		Secondary outcome measure	Secondary outcome results		Results summary
			Before treatment	After treatment		Before treatment	After treatment	
1	Parreira da Silva et al. (2020) [11]	N/A	N/A	N/A	Occlusal force	Baseline: C = 518N, T = 468N	1 month: C = 355N, T = 479N	BoNT-A resulted in a clinically significant decrease in occlusal force and muscle hyperactivity in patients with TMD. The biting force was lowest at 1 month with a transient increase up to 6 months. The activity in the left and right masseter muscles decreased significantly from baseline at 1 month and increased up to 6 months. However, the EMG measure did not reach the baseline value at 6 months.
2	Sitnikova et al. (2022) [12]	N/A	N/A	N/A	EMG	Baseline: T = 222 mV, C = 155 mV	2 weeks: T = 62 mV, C = 158 mV	A significant decrease in muscle activity was seen at 2 weeks in the BoNT-A group. Subsequently, the EMG measure transiently increased up to 32 weeks crossing the baseline activity. Occlusal activity in the BoNT-A group was the lowest at 2 weeks, reached baseline at 27 weeks, and was significantly higher than baseline at 32 weeks.
3	Shim et al. (2020) [13]	N/A	N/A	N/A	EMG	MVC MA (μV)	MVC MA (μV)	BoNT-A effectively reduced muscle hyperactivity in patients with TMD. The improvements were highest at 1 month at remained stable at 3 months.
4	Zhang et al. (2016) [14]	N/A	N/A	N/A	Occlusal force	N/A	1 month: T = −41.97 (9.55), P= −7.97 (6.87), C= 0.94 (6.90)	BoNT-A significantly reduced occlusal force in patients with TMD at 1, 3, and 6 months.
5	Gupta et al. (2016) [15]	Pain	Baseline: T= 11.25, C= 9.08	At 2 weeks: T= 5.25, C=6.67	EMG changes in RMR: Right masseter region(RMR) and Left masseter region(LMR)	Baseline: RMR: 93.67 (56.19) LMR: 104.5 (98.89)	2 weeks: RMR: 29.08 (16.56) LMR: 30.83 (37.00)	BoNT-A can significantly reduce pain when compared to pain reduction in the placebo group when measured at 2 weeks and 1 month. There was a significant decrease in muscle activity at 2 weeks and 1 month.
6	De la Torre Canales et al. (2020) [16]	Pain	N/A	2 weeks: Group A: significant reduction, Group B: significant reduction, Group C: significant reduction, C: no significant reduction	EMG changes	N/A	1 month: Group A: significant reduction, Group B: significant reduction, Group C: significant reduction, C: insignificant reduction	BoNT-A can effectively reduce persistent myofascial pain irrespective of the doses administered when compared to placebo. EMG activity decreased at 1 month but recovered at 3 months in all doses
7	Kurtoglu et al. (2008) [17]	Pain	At Baseline: C = 58.9 T = 56.1	2 weeks: C = 51.1, T = 45.8	EMG changes	Baseline: C = 200.0/529.3. T = 206.3/296.0	2 weeks: C = 252.3/498.8, T = 165.0/199.0	The pain significantly lowered at 2 and 4 weeks. There was a decrease in masseter muscle activity at 2 weeks, which improved by the 4th week.
8	Ernberg et al. (2001) [18]	Pain	baseline: C = 54 T = 58	1 month: C = 27, T = 35 3 months: C = 24 T = 34	Maximum mouth opening (mm)	baseline: C = 43.4 T = 42.7	1 month: C = 44.3, T = 44.3	No significant difference in the pain parameters and jaw opening was reported post-BoNT-A injections at 1 and 3 months.
9	De Carli et al. (2016) [19]	Pain	Baseline: C = 7, T = 7	1 month: C = 3.5, T = 3	Maximum mouth opening (mm)	Baseline: C = 42, T = 38	1 month: C = 42, T = 36	Laser therapy and BoNT-A effectively reduced pain at 1 month. Laser therapy reduces pain earlier than BoNT-A. No statistically significant changes in the parameters of jaw opening were observed in both interventions.
10	Nixdorf et al. (2002) [20]	Pain	N/A	2 months: C = reduced by 1 mm, T = reduced by 19 mm	Maximum mouth opening (mm)	N/A	2 months: C = increased by 5 mm, T = decreased by 3 mm	Pain intensity did not show any significant decrease after treatment with BoNT-A as assessed at 2 months. Maximum mouth opening decreased in the BoNT-A cohort at 2 months.
11	Guarda-Nardini et al. (2008) [21]	Pain	Baseline: T= 5.00, C= 3.90	1 week: T= 4.60, C= 3.00	Maximum mouth opening (mm)	Baseline: T= 50.70, C= 48.00	1 week: T= 51.00,C= 47.30	The maximum mouth opening increased and the pain score reduced significantly in the BoNT-A group at 1 week, 1 month, and 6 months.
12	Guarda-Nardini et al. (2012) [22]	Pain	Baseline: T= 7.3 (1.1), C= 6.0 (2.0)	3 months: T= -2.5, C= -3.5	Maximum mouth opening (mm)	Baseline: T= 48.7 (8.3), C= 52.0 (9.5)	3 months: T= +2.7, C= +0.4	Both BoNT-A and fascial manipulation cause a significant decrease in pain levels at a 3-month follow-up. Fascial manipulation is superior in the reduction of subjective pain than BoNT-A. BoNT-A injections improve the jaw's range of motion.
13	Kim et al. (2023) [23]	Pain	Baseline: T= 5.00 ± 1.45, C= 5.36 ± 1.44	1 month: T = 2.64 ± 2.02, C= 3.64 ± 2.06	Maximum mouth opening (mm)	Baseline: T= 46.57 ± 7.18, C= 48.00 ± 6.61	1 month: T = 45.71 ± 7.96, C= 47.00 ± 5.72	BoNT-A reduced pain level compared to baseline and placebo but reported no significant effect on maximum mouth opening at 1, 2, or 3 months.
14	Kütük et al. (2019) [24]	Pain	Baseline: T= 5.3±1.7, C= 5.4±1.7	1.5 months: T= 4.2±1.4, C= 3.1±1.8	Maximum mouth opening (mm)	Baseline: T= 42.8±5.0, C= 42.2±5.8 0.708	1.5 months: T= 43.7±5.0, C= 45.0±5.8	BoNT-A and dry needling cause a significant reduction in pain levels and a statistically significant increase in the maximum mouth opening measure at 1 month.
15	Montes-Carmona et al. (2020) [25]	Pain	Baseline: C= 6.47 (± 0.96) LD= 6.45 (± 1.09), T= 6.5 (± 0.94)	1 week: T = 4.95 (1.27), C = 5.42 (1.16), LD = 6.55 (1.05)	Maximum mouth opening (mm)	Baseline: C= 43.05 (± 6.88) LD= 43.35 (± 5.18), T= 40.7 (± 5.43)	1 week: T = 41.55 (5.15), C = 43.05 (7.09), LD = 43.25 (4.78)	BoNT-A demonstrated a statistically significant reduction in pain levels and an increase in maximum mouth opening compared to both saline and lidocaine controls at 1, 2, 4 weeks, and 2, 3, and 6 months.
16	Ângelo et al. (2023) [26]	Pain	Baseline: T= 5.4(1.9), C= 6(3.6)	At 5 weeks: T= 0.9(1.3), C= 0.9(1.6)	Maximum mouth opening (mm)	Baseline: T= 35.1(12.8), C= 33.9(10.1)	At 5 weeks: T= 36.3(3.3), C= 35.9(2.2)	At both the timepoints of 5 weeks and 6 months, pain reduced and maximum mouth opening increased significantly in BoNT-A as well as the placebo group. Intragroup differences between placebo and BoNT-A injection were not statistically significant.
17	Rezazadeh et al. (2022) [27]	Pain	Baseline: T = 4.72, C = 3.50	1 week: T = 2.11, C = 1.78	Maximum Opening (mm)	Baseline: T = 43.45, C = 49.82	1 week: T = 43.89, C = 47.27	Evaluation of maximum mouth opening in the BoNT-A group revealed no statistically significant changes at 1 week, 1 month, and 3 months. No significant differences were observed between the BoNT-A and placebo groups concerning maximum mouth opening. A significant reduction in pain was reported at 1 week following BoNT-A, with stable pain reduction measured at three months.
18	Jadhao et al. (2017) [28]	Pain	Baseline: T = 3.8 (1.13), P= 4 (0.8), C= 4 (0.9)	1 week: T = 3.55 (1.19), P= 3.85 (0.88), C= 3.80 (0.9)	Occlusal force		Change from baseline	BoNT-A significantly reduced pain and occlusal force as compared to patients with placebo or no treatment provided as assessed at 1 week, 3 months, and 6 months.
19	Patel et al. (2017) [29]	Pain score (0-10)	Baseline : C = 5.43, T = 5.4	1 month: C = 3.72, T = 0.9	N/A	N/A	N/A	A clinically significant reduction in pain levels in the BoNT-A group was observed at 1, 2, and 3 months.
20	Ondo et al. (2018) [30]	Pain	N/A	1 month C= 44.2 ± 14.3, T= 65.0 ± 19.6	N/A	N/A	N/A	A clinically significant reduction in pain levels in the BoNT-A group was observed at 1-month follow-up.
21	Von Lindern et al. (2003) [31]	Pain	N/A	1 month: T= Baseline - 3.2, C= Baseline - 0.4	N/A	N/A	N/A	A clinically significant reduction in pain levels in the BoNT-A group was observed at 1-month follow-up. It reduced pain in up to 90% of patients who have failed to achieve adequate relief with conventional treatment strategies.
22	Cruse et al. (2022) [32]	Pain	Baseline: 2.93(1.28)	1 month: T= 2.25(1.11), C= 2.48(1.39)	N/A	N/A	N/A	No significant decrease in pain was observed at 1 and 3 months in BoNT-A and placebo groups.
23	Al-Wayli H (2017) [32]	Pain	Baseline: T= 7.1 ± 0.72, C= 7.5 ± 0.66.	3 weeks: T = 4.6 ± 0.58, C = 5.4 ± 0.58	N/A	N/A	N/A	A clinically significant reduction in pain levels in the BoNT-A group was observed at 3 weeks, and 2, 6, and 12 months.

The primary outcome of the current study was to assess the analgesic effect of BoNT-A injection on facial muscles and reduce pain in patients with TMD. The visual analog scale (VAS) was the primary pain assessment tool. Other outcome measures included in the study were self-reported questionnaires [15,17], pain scores [29], and short-form McGill pain scores [32].

Out of 19 studies assessing pain levels after injecting BoNT-A, six reported a significant decrease in pain scores in patients injected with BoNT-A compared with the placebo cohort [15-17,21,29,33], providing evidence for the analgesic properties of BoNT-A. Conversely, four studies showed mitigation of pain levels in both the intervention and placebo groups. In four studies, there was no statistically significant difference between the pain levels of the two groups, suggesting a similar analgesic effect of the placebo and BoNT-A [18,20,26,27]. Jadhao et al. (2017) found a significant decrease in VAS scores following BoNT-A treatment compared with both the placebo and no-treatment groups [28].

One study found no improvement in pain levels after BoNT-A administration. The study suggested the use of higher doses and increasing injection points to achieve optimal pain reduction [32]. Another study aimed to identify the dose-dependent response of BoNT-A in patients with chronic myofascial pain, but found no correlation between the analgesic properties and the dose of BoNT-A. However, Kim et al (2023) administered high doses of BoNT-A (150 units on each side) and reported a clinically significant reduction in VAS scores [23]. Similarly, Ondo et al. (2018) reported pain mitigation at high doses of 100 units of BoNT-A on each side [30].

Several studies have compared BoNT-A therapy with other therapeutic options for the management of TMD. Von Lindern et al. (2003) assessed pain reduction in patients who had not received pain relief from conventional treatments and found that BoNT-A successfully reduced pain in 90% of these patients [31]. By comparing the pain reduction efficiency of BoNT-A with laser therapy, De Carli et al. (2016) found that while laser therapy provided faster pain reduction, BoNT-A resulted in a significantly greater reduction in pain levels [19]. A comparative analysis of the efficiency of BoNT-A and fascial manipulation in mitigating myofascial pain revealed a greater decrease in VAS score in the fascial manipulation cohort [22]. Another study reported superior pain reduction with dry needling compared to BoNT-A injection [15]. Montes-Carmona et al. (2020) compared BoNT-A to another pharmacological agent, lidocaine [25]. BoNT-A reduced pain levels transiently for up to six months, whereas the lidocaine group observed no significant changes in the VAS score.

Secondary Outcomes

The occlusal force of the posterior teeth measured during mastication has been assessed in four studies [11,12,14,28]. All four studies reported a decrease in the maximum biting force when BoNT-A was injected. Jadhao et al. (2017) compared BoNT-A with placebo and in patients with no treatment provided [28]. The maximum decrease in biting force was observed in the BoNT-A cohort. The placebo group showed a statistically significant decrease, but it was much lower than that of BoNT-A. In patients without medical intervention, minimal biting force changes were observed [28]. Two studies reported a decrease in occlusal force that partially recovered over six months [11,14]. Only one study observed the occlusal force making a full recovery to the baseline value at 27 weeks and an improvement in the occlusal force at 32 weeks [12].

EMG measurements provided evidence of changes in facial muscle activity. Kurtoglu et al. (2008) observed an initial decrease in EMG activity at two weeks, which improved significantly by one-month post-BoNT-A injection [17]. Similarly, another study observed that EMG activity decreased when measured at an interval of one month but recovered at three months, irrespective of the doses [16]. Gupta et al. (2017) found a statistically significant decrease in the left and right masseter muscle activity at two weeks and after one month [15]. Two studies reported a reduction in EMG measures during the initial follow-up period and showed improvement at long-term assessments [11,12]. One study suggested a long-term decrease in muscle activity [13].

Three studies have suggested an increase in the range of mandibular movement and mouth-opening capacity following BoNT-A injection into the masticatory muscles [21,24,25]. Ângelo et al. (2023) reported an increase in mandibular movement in both the intervention and placebo groups, with no significant intragroup difference [26]. A study comparing changes in jaw movement between two techniques, BoNT-A therapy, and laser therapy, showed no increase in maximum mouth opening in both interventions [19]. Three studies reported a statistically insignificant increase in the maximum mouth-opening capacity of patients in either the BoNT-A or placebo group when assessed between one and three months [18,23,27]. Conversely, one study reported a decrease in jaw opening after employing BoNT-A [20].

Risk of Bias Assessment

The quality of the studies was evaluated using the Cochrane Risk of Bias (RoB 2.0; Cochrane, London, UK). The assessment summary is presented in Table 4.

**Table 4:** Critical appraisal of randomized controlled trials

		Domains					
Sl. No.	Author (year)	D1 (randomization process)	D2 (deviations from the intended interventions)	D3 (missing outcome data)	D4 (measurement of the outcome)	D5 (selection of the reported result)	The overall risk of bias
1	Parreira da Silva et al. (2020) [11]	Low	Low	Low	Some concerns	Some concerns	Low
2	Sitnikova et al. (2022) [12]	Low	Low	Low	Low	Low	Low
3	Shim et al. (2020) [13]	Some concerns	Low	Low	Low	Low	Low
4	Zhang et al. (2016) [14]	Some concerns	Low	Low	Some concerns	Some concerns	Some concerns
5	Gupta et al. (2016) [15]	Some concerns	Low	Low	Low	Some concerns	Low
6	De la Torre Canales et al. (2020) [16]	Low	Low	Low	Low	Low	Low
7	Kurtoglu et al. (2008) [17]	Low	Some concerns	High	Some concerns	Some concerns	Some concerns
8	Ernberg et al. (2001) [18]	Low	Low	Low	Some concerns	Some concerns	Low
9	De Carli et al. (2016) [19]	Low	Low	High	Low	Some concerns	Low
10	Nixdorf et al. (2002) [20]	Low	Low	High	Low	Some concerns	Low
11	Guarda-Nardini et al. (2008) [21]	Some concerns	Low	Some concerns	Low	Some concerns	Some concerns
12	Guarda-Nardini et al. (2012) [22]	Some concerns	High	Low	Low	Some concerns	Some concerns
13	Kim et al. (2023) [23]	Low	High	Some concerns	Low	Some concerns	Some concerns
14	Kütük et al. (2019) [24]	Low	Some concerns	Low	Low	Some concerns	Low
15	Montes-Carmona et al. (2020) [25]	Low	Some concerns	Low	Some concerns	Some concerns	Some concerns
16	Ângelo et al. (2023) [26]	Low	Low	Low	Low	Low	Low
17	Rezazadeh et al. (2022) [27]	Low	Some concerns	Low	Low	Low	Low
18	Jadhao et al. (2017) [28]	Some concerns	High	Some concerns	Some concerns	Some concerns	Some concerns
19	Patel et al. (2017) [29]	Low	Some concerns	Low	Low	Some concerns	Low
20	Ondo et al. (2018) [30]	Low	Some concerns	Low	Some concerns	High	Some concerns
21	Von Lindern et al. (2003) [31]	Some concerns	Low	Some concerns	High	Some concerns	Some concerns
22	Cruse et al. (2022) [32]	Low	Low	Low	Low	Low	Low
23	Al-Wayli H (2017) [33]	Some concerns	Low	Low	Low	Some concerns	Low

Risk assessment was conducted for the five domains as follows. Randomization: low risk in 15 studies, some concerns in eight; Deviations from Intended Interventions: low risk in 14 studies, moderate in six, high in three; Missing Data: low risk in 16 studies, moderate in four, high in three; Inaccurate measurement: low risk in 15 studies, moderate in seven, high in one; Selection of the Reported Results: low risk in six studies, moderate in 16, high in one. The overall risk of bias was low in 14 studies and nine had some concerns.

Discussion

The primary aim of this study was to assess the changes in myofascial pain in patients with TMDs who received BoNT-A injections. In the included 23 RCTs, the study population included 750 patients who had TMDs. A total of 454 participants were injected with BoNT-A; 313 were administered a placebo (saline), 25 underwent dry needling, 20 were administered lidocaine, and 48 received non-pharmacological treatments. The secondary outcomes evaluated were maximum mouth opening, changes in biting force, and changes in EMG measurements.

This study provides evidence supporting the effectiveness of BoNT-A therapy in reducing pain in patients with TMDs. The exact mechanisms underlying the reduction of pain in myofascial regions by BoNT-A are unclear. However, evidence suggests a significant role for both peripheral and central nervous system mechanisms in the pathogenesis of pain in disorders of the TMJ [31,34,35]. Treatment of TMD with BoNT-A has emerged as a promising therapeutic strategy due to the documented analgesic effects on TMJ that occur through several mechanisms. The primary mechanism involves direct signal reduction in the facial muscles by localized chemical denervation of neurons in the TMJ [9]. BoNT-A inhibits the release of acetylcholine at the neuromuscular junction and prevents muscle fiber activation. Furthermore, BoNT-A exerts a direct analgesic effect by partially antagonizing the release of specific neurotransmitters involved in nociception, such as calcitonin gene-related peptide (CGRP) and glutamate [9].

The onset of this analgesic effect usually occurs within a few days following BoNT-A injection [21]. The pain reduction, however, is prolonged and sustained in the long term for up to six months. The extended duration of action of BoNT-A for pain management, up to six months post-injection, might be attributed to the unique properties of its protease. A toxin evades the degradation pathways of cellular protease and persists within the cell cytoplasm for a prolonged period [36]. When the cell cannot effectively degrade BoNT-A, the therapeutic effects remain stable.

Facial manipulation therapy demonstrated an immediate reduction in VAS pain scores compared with BoNT-A treatment [22]. However, this difference was not statistically significant at the three-month follow-up, with BoNT-A providing a greater reduction in pain levels [22]. Several factors can explain this initial disparity. Facial manipulation therapy involves multiple sessions (3±1) compared with a single BoNT-A injection [37]. Furthermore, the therapist's application of deep digital pressure during the 50-minute facial manipulation sessions could have exerted a positive psychological influence [37]. Mental relaxation during the procedure potentially fostered a positive therapeutic relationship and contributed to pain reduction. This is in contrast with the delayed onset of action of BoNT-A, whose effects typically manifest several days after injection [21]. Therefore, immediate BoNT-A efficacy assessments are not directly comparable to those of other therapies. For accurate evaluations, studies must focus on medium and long-term outcomes to capture the cumulative effect of BoNT-A treatment [37].

Similar findings were observed when comparing low-level laser therapy with BoNT-A, suggesting the potential advantage of immediate pain relief with manual therapies [19]. Dry needling also alleviates myofascial pain at rest and during chewing [24]. These non-pharmacological interventions have higher acceptability due to their non-invasiveness and absence of chemical use. The pharmacological treatment options discussed in this systematic review were lidocaine injections and the dry needling technique. Patients treated with lidocaine reported significantly inferior clinical outcomes compared with BoNT-A treatment [25]. Lidocaine injections are a commonly employed approach for managing TMD, yet they demonstrated a lack of statistically significant pain reduction in all follow-up periods. However, previous clinical studies have reported contradictory findings, with lidocaine improving pain in facial muscles [38]. Lidocaine inhibits nerve conduction by blocking sodium channels [39,40]. The inhibition of transfer through the sodium channels produces analgesic effects in facial muscles and suppresses hyperexcitability [38].

The current study showed the efficacy of BoNT-A in improving jaw function, as assessed by maximum mouth opening. Previous systematic reviews have reported similar inconsistencies in determining the role of BoNT-A in increasing the jaw range of motion [34,41]. The rationale behind the variation in results is the limitation of the diagnostic criteria of the included studies. Restriction of mouth opening is commonly associated with TMDs such as articular disc displacement and osteoarthritis. However, most of the included studies focused on patients with myofascial pain. Unlike the aforementioned TMDs, myofascial pain often does not involve concomitant joint dysfunction thereby preserving normal functional mobility in the jaw. Limited jaw mobility was not a diagnostic criterion in any of the studies. Emberg et al. (2001) observed no statistically significant difference in maximum mouth opening between the control and BoNT-A-treated groups [18]. However, the initial mouth opening was near the normal maximum mouth opening in the general population. Therefore, an increase in maximum mouth opening may not be possible, regardless of treatment. To definitively assess the role of BoNT-A in improving jaw mobility, future studies should include participants with a primary complaint of limited maximum mouth opening in comparison with a placebo control group. This approach can provide more robust evidence for the clinical application of BoNT-A injection in managing jaw mobility in patients with myofascial TMD.

The occlusal force decreased in all the studies. This was an expected outcome of the study, as the decrease in the maximum biting force after BoTN-A injection was in line with the pharmacokinetic mechanism of action. The therapeutic effect of BoNT-A is manifested by the denervation of the injected sites. Alterations in the signaling pathways of motor neurons result in impaired movement of the masseter muscle [12]. Because the masseter is an important muscle used for biting, its denervation directly affects the occlusal force [14]. The recovery of occlusal force varied across the included studies, with some reporting partial recovery and others reporting complete recovery. This variation was attributed to the doses used. The definitive impact of BoNT-A on occlusal force loss requires standard doses to determine this latent adverse effect [12].

This systematic review reported a decrease in EMG activity. BoNT-A reduces pain in patients with TMD by blocking synapses between neurons. This is the primary rationale for the decrease in EMG measurements [42]. The EMG activity when assessed after prolonged duration, showed recovery. This provides evidence of the formation of new synaptic junctions and a reduction in the effect of BoNT-A after six months.

The heterogeneity in study outcomes is an important concern. While BoNT-A showed efficacy in reducing pain in many studies, others reported mixed results, particularly in secondary outcomes such as maximum mouth opening, occlusal force, and EMG changes. Some studies reported significant improvements in maximum mouth opening, while others found no significant difference compared to placebo [17]. This variation could be due to the initial mouth-opening measurements, patient characteristics, or differences in study methodologies.

The VAS score was the primary method used to assess changes in pain perception. Most studies have evaluated pain using this self-reported technique. Although VAS is widely used, there is uncertainty about its accuracy in pain assessment. VAS scores are inherently subjective, introducing variability in pain assessment due to factors such as data collection methods and recall bias. Studies have suggested that VAS is a single-time, dependent measure that relies heavily on patient recall [41]. Recalling a patient’s pain is prone to significant variation, resulting in inaccurate pain evaluation [43]. Conversely, Conti et al. (2001) compared the reliability and validity of a behavioral scale to VAS. Their findings suggested that a numerical rating scale has superior validity in capturing reproducible pain scores [44]. This highlights the need for a quantitative measure of pain rather than subjective techniques.

A diverse experimental protocol was used in these studies. The dose variation ranged from 50 to 300 units. Along with the differences in doses, the sites of injection were different muscles, with variations in the number of injected points. There is a lack of consensus regarding the accepted standard dose of BoNT-A [45]. Although the Food and Drug Administration (FDA) recommends the use of 400 units of BoNT-A within three months to be safe, all doses were decided as per the study requirement for treating TMD. However, an effective evaluation of study outcomes requires the use of the lowest effective dose that is safe and prevents the formation of antibodies and poisoning from overdosage [45].

Several studies included in this systematic review defined Diagnostic Criteria for TMD (DC/TMD). A study conducted by Schiffman et al. (2014) reported the critical need for standardizing TMD diagnosis by proposing a novel Diagnostic Criteria for TMD framework [46]. This framework incorporates a validated screening tool for the initial detection of pain associated with TMD, followed by specific diagnostic criteria to differentiate the most prevalent TMD subtypes. The DC/TMD protocol is supported by robust evidence and is applicable in both clinical and research settings. Standardized instruments within this framework facilitate accurate identification of a broad spectrum of TMD presentations across diverse patient populations. However, only five out of the 23 studies employed DC/TMD [12,21-23,26], and six employed the Research Diagnostic Criteria for TMD [16-18,20,25,27] for diagnostic purposes.

Some limitations and probable biases must be considered when interpreting the results of this systematic review. First, the heterogeneity among the included studies, particularly in terms of study design, dose of BoNT-A used, and injection techniques, presents inconsistency in the outcomes. Many studies did not follow a standardized treatment protocol, with BoNT-A doses ranging from 50 to 300 units and varying injection sites. This lack of standardization makes it difficult to draw definitive conclusions regarding the optimal dosage and injection technique for BoNT-A in TMD treatment. Future studies should aim to establish standardized treatment protocols to improve comparability across trials.

Additionally, only a small group of the included studies used the Diagnostic Criteria for TMD (DC/TMD), which is considered the gold standard for TMD diagnosis [45]. The lack of consistent use of validated diagnostic tools like DC/TMD or Research Diagnostic Criteria for TMD (RDC/TMD) limits the generalizability of the findings and introduces potential bias in patient selection. Future research should emphasize the importance of using standardized diagnostic criteria to ensure that study populations are comparable and that treatment effects are accurately measured.

The findings of this systematic review have important suggestions for clinical practice. While BoNT-A appears to be an effective treatment option for reducing pain and muscle activity in patients with TMDs, clinicians must be aware of its possible adverse effects, including decreased occlusal force and muscle weakness. Standardized treatment protocols, including guidelines on dosing, injection site, and frequency, are needed to minimize these adverse outcomes and optimize patient care. Clinicians should also consider the delayed onset of BoNT-A’s effects and its role in long-term pain management, especially when other treatments offer instant relief.

Strengths of the study

This systematic review analyzed data explicitly from RCTs. Numerous studies were double-blind to eliminate bias in the evaluators. RCTs have the highest quality of evidence and minimum bias in the confounding factors. Randomization of participants ensured fair allocation of patients into respective cohorts. A controlled design further improves the quality by removing inherent biases. This study also incorporated the PRISMA protocol for study selection. The Cochrane Risk of Bias tool was used to assess the quality of the study. The number of patients included in this review was significantly large, improving the study results' quality.

Limitations of the study

Some limitations of this systematic review must be considered. The study methodologies and outcome measurements were heterogeneous among the included studies. The study's experimental protocol included diverse BoNT-A doses, injection sites, follow-up intervals, participant characteristics, and predispositions toward female patients. This heterogeneity poses a significant challenge compared to extracting uniform results, which reduces the generalizability of the results. The study did not include a meta-analysis because of the diverse outcomes reported and the variations in protocols and follow-up periods. Secondary outcomes were reported in a limited number of studies for which meta-analysis could not be performed. Studies included also patients who received conventional treatments before BoNT-A injections for TMD but did not achieve success. Such cases may represent a population of complicated TMDs that cause unfavorable outcomes for BoNT-A. Numerous studies included in this review did not disclose conflicts of interest or funding sources, which could lead to potential bias. Studies sponsored by pharmaceutical companies, including those making BoNT-A, may be more likely to report positive outcomes. On the contrary, studies funded by independent organizations or government grants might offer a less subjective perspective. Future studies should prioritize transparency in reporting funding sources and potential conflicts of interest to guarantee unbiased results. Furthermore, disclosure of the authors’ connections with any companies involved in producing or promoting BoNT-A is fundamental for assessing potential biases in the study outcomes.

## Conclusions

This study provides evidence supporting the usefulness of BoNT-A in reducing pain in patients with TMDs. The muscle activity and biting force were significantly reduced in the therapeutic groups. However, the heterogeneity in study outcomes, the lack of standardized treatment protocols, and the inconsistencies in diagnostic criteria highlight the need for further research to establish the optimal use of BoNT-A in clinical practice. Clinicians should carefully weigh the benefits and risks of BoNT-A therapy, considering the long-term analgesic effects and potential adverse outcomes, to provide evidence-based care for patients with TMDs. Specification of the injection site, depth, interval between injections, and dose is necessary to improve the patient care regime. Future studies should explore the long-term effects of BoNT-A on jaw function, particularly in patients with limited maximum mouth opening, to better understand its role in improving functional outcomes.
